# Deep neural networks reveal topic-level representations of sentences in medial prefrontal cortex, lateral anterior temporal lobe, precuneus, and angular gyrus

**DOI:** 10.1016/j.neuroimage.2022.119005

**Published:** 2022-02-14

**Authors:** David J. Acunzo, Daniel M. Low, Scott L. Fairhall

**Affiliations:** a CIMeC/University of Trento, Corso Bettini 31, Rovereto 38068, Italy; b Program in Speech and Hearing Bioscience and Technology, Harvard Medical School, United States; c Brain and Cognitive Sciences Department, MIT, United States

**Keywords:** Representational similarity analysis, Convolutional neural network, fMRI, Sentence processing, Semantic representation, Semantic system

## Abstract

When reading a sentence, individual words can be combined to create more complex meaning. In this study, we sought to uncover brain regions that reflect the representation of the meaning of sentences at the topic level, as opposed to the meaning of their individual constituent words when considered irrespective of their context. Using fMRI, we recorded the neural activity of participants while reading sentences. We constructed a topic-level sentence representations using the final layer of a convolutional neural network (CNN) trained to classify Wikipedia sentences into broad semantic categories. This model was contrasted with word-level sentence representations constructed using the average of the word embeddings constituting the sentence. Using representational similarity analysis, we found that the medial prefrontal cortex, lateral anterior temporal lobe, precuneus, and angular gyrus more strongly represent sentence topic-level, compared to word-level, meaning, uncovering the important role of these semantic system regions in the representation of topic-level meaning. Results were comparable when sentence meaning was modelled with a multilayer perceptron that was not sensitive to word order within a sentence, suggesting that the learning objective, in the terms of the topic being modelled, is the critical factor in capturing these neural representational spaces.

## 1. Introduction

The human brain represents concepts not only as single elements but combines them to form more complex ideas. While single concepts can be well represented by individual words, such as *dog* or *release*, the combination of these concepts into more complex pieces of information and knowledge is, in part, reflected in the information contained in sentences. Within a sentence, constituting words provide context to and inform each other to facilitate the comprehension of the often complex and specific meaning that it conveys. The higher level meaning in sentences can convey meaning that relates not only to single concepts but to general topics of information, for example, the topics ‘religion’ or ‘sports’. Consequently, understanding the neural processes associated with the higher, topic-level, meanings of a sentence may be facilitated by using models that specifically aim to capture these topic-level meanings and that represent word meaning within the context of the sentence, rather than representing those words as decontextualized elements.

content of single concepts (Bruffaerts et al., 2013 Devereux et al., 2013; Fairhall and Caramazza, 2013; Liuzzi et al., 2020;).

The processing of sentences has been investigated using univariate techniques, for instance contrasting the response to sentences to lists of non-words or scrambled sentences (Brennan and Pylkkänen, 2012 Fedorenko et al., 2010; E. 2016;) or identifying parametric increases in activity with the number of linguistic constituents within a series of words or pseudowords (Pallier et al., 2011). This comparison aims to identify not just the semantic meaning of the sentence (i.e. the end result: the sentence-level meaning) but also the integrative linguistic processes through which words are combined (the process of sentence comprehension). While the regions identified in these studies largely overlap with the left-lateralized semantic system described by Binder and colleagues (Binder et al., 2009), they additionally include the middle and superior frontal gyri and do not involve the dmPFC, vmPFC or precuneus (Fedorenko et al., 2010 Hagoort, 2016;).

In the present study, we are interested in uncovering brain areas that hold representations of the semantic context or topic at the level of sentences. Recent advances in machine learning provide a potential tool for investigating human topic-level representations. Topic modelling is indeed an established field in text mining, whose goal is to develop techniques to automatically find structures and annotate text data, and has been used in a wide range of fields ranging from natural language processing, software engineering, bioinformatics and the humanities (Kherwa and Bansal, 2018 Vayansky and Kumar, 2020;). Human topic processing has mostly been investigated in the context of discourse comprehension, most particularly studying inferences (how we link the meaning of sentences or paragraphs together) or integration (how we process discourse units within their more general context; i.e. a sentence within a paragraph or a paragraph within a story (Regev et al., 2013 Egidi and Caramazza, 2013;; see Yang et al., 2019 for a recent meta-analysis). Results points toward an involvement of regions belonging to the semantic network, in particular the bilateral IFG, the dmPFC and the MTG. On the other hand, studies comparing sentences drawn from a limited number of distinct experimenter-defined topics based on specific object classes (e.g. sentences related to people, food, places or objects) have found the activation of distributed networks that were selective for these different domain-based topics (Rabini et al., 2021).

We approached the problem of constructing sentence topic-level representations by focusing on a Convolutional neural network (CNNs). This architecture was selected as CNNs create representations of sentences through the hierarchical combination of tokens into successively larger chunks and units of semantic meaning. CNNs trained under supervised learning have enjoyed great success in the field of computer vision (e.g Krizhevsky et al., 2012.) and successfully been used to classify sentences and achieve a classification accuracy comparable to recurrent neural networks, which are built upon an architecture more closely following the left-right process of sentence construction (Zhang and Wallace, 2017). Using sentence topics as training categories, the sentence representations extracted from such CNNs can then be used as a model to capture the added meaning arising from combination of words within a sentence, which in turn can be compared to neural patterns measured with brain imaging.

In this study, we sought to identify where sentence topic-level representations are most strongly represented in the brain compared to its constituent conceptual units (words). This approach is largely distinct from approaches whose aim is to understand the linguistic processes through which meaning is constructed, i.e. the syntactic combination of words. This is in particular the focus of studies looking for the neural bases of Merge-like processes combining words into hierarchical structures (Zaccarella et al., 2017), or for the progressive formation and update over time of integrated meaning (conceptual ‘gestalt’; (Branzi et al., 2020). We instead employ of a computational model that extracts thematic consistencies between words to generate a sentence topic-level representation that we then compare to neural representations.

We trained a CNN to categorize sentences from Wikipedia, the largest text corpus of human knowledge, into 64 broad semantic categories or topics. Rather than parsing sentences through syntactic rules, the CNN learns by performing local-to-global (from trigram to whole sentence) operations. In that way, it flexibly constructs combinatorial meaning at progressively large scales of meaning. We demonstrate that the last layer of this CNN provides a representation of the overall meaning of the sentence, which we then used to construct a sentence topic-level representational semantic space. We refer to this as topic-level sentence meaning as the representation reflects information both about the localised context of words within the sentence and about the topics being modelled. These topic-level sentence representations were contrasted to context-free word-level sentence representations using an averaged word embedding model. We refer to these as word-level representations as they model the words independently both of their present context and of the topics being modelled. We then recorded participants’ brain responses while reading sentences using fMRI. By applying representational similarity analysis (RSA), we uncovered which cortical regions more fully captured the topic-level sentence meaning than the word-level meaning. In so doing, we can identify neural systems involved in the high-level representation of sentence-level meaning more than the constituent words themselves.

## 2. Materials and methods

### 2.1. Semantic models design

#### 2.1.1. Convolutional neural network design and training

The dataset was composed of Wikipedia sentences labelled according to the category (or topic) of the article to which they belong. For this purpose, we used the DBpedia project (Lehmann et al., 2015), which links each one of the Wikipedia articles (1.4 million in Italian) to a category within an ontology (e.g., *Airport, Mountain, Politician, Tennis Player, Musical Artist*). The sentences of every Italian Wikipedia article were thus tokenized (the samples) and linked to their articles’ DBpedia category (the labels). Since some categories have very few articles, 64 categories were chosen from the 320 categories available to obtain similar sample sizes of 6000 sentences per category between 6 and 38 words. Sentences were pre-processed so that all punctuation was removed and digits replaced by the hash character ‘#’. An 80–20 train-test split was applied which resulted in a training set of 4800 sentences per category used for hyperparameter tuning through 5-fold cross validation and a test set of 1200 sentences per category.

**Fig. 1.**
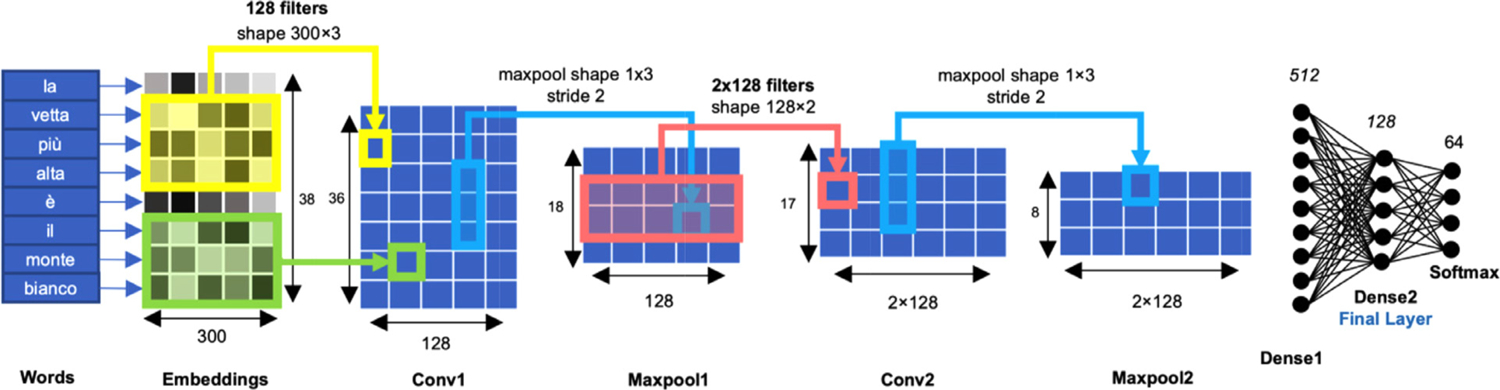
Convolutional neural network (CNN) architecture. The architecture comprises two convolutional-maxpool layers followed by two dense layers. The input (sentence) is a list of word tokens zero-padded to 38, and the output is a vector of size 64 corresponding to the prediction probability that the input sentence belongs to each of the 64 categories. The first layer assigns an embedding vector of size 1 × 300 to each word, constituting a 38 × 300 sentence matrix. Conv1 is the first convolutional layer applying 128 filters of weights to the preceding layer through a dot product resulting in a single value per filter and trigram. Each filter can be seen as a sliding window looking at one trigram at a time applying a dot product between the trigram values and the filter weights. The filter weights are learned by the model in such a way to optimize the classification of the different categories and initialised using a random seed. Maxpool1 extracts the maximum value from every 3 values of Conv1, keeping only the largest features values. These Conv-Maxpool layers are repeated for gradual composition, and are then followed by two fully-connected dense layers which allow the information of the whole sentence to be accessed regardless of word order. Finally, information is passed through a softmax layer which is a probability distribution summing to 1 and assigns a probability to 64 possible categories, the highest of which becomes the model’s prediction. ‘Dense 2 ′ (Final Layer) is the layer used in the present study to build the sentence-level representational space (see Fig. 2).

We designed an architecture with a trainable embedding layer, two convolutional-maxpool layers, two fully connected layers and a softmax layer (see Fig. 1) (for further discussion, see Zhang and Wallace, 2017). Sentences were fed to the network as a list of zero-padded 38 word tokens. Embedding values were initialised using Italian fastText word embeddings (Grave et al., 2018). The CNN was built with Keras (https://keras.io). Gridsearch hyperparameter tuning and model training were performed on the GPU Peregrine HPC cluster of the University of Groningen and the Titan V GPU of the University of Trento donated by NVidia Corporation.

After hyperparameter tuning, the final parameters of the CNN were: dropout rate: 0.2, batch size: 512; optimizer: Adam; activation: ELU; number of filters: 128, and performance (both validation loss and accuracy) stopped improving at 3 epochs, so epoch was set at 3 to avoid over-fitting. Training cross-validation accuracy was 68.65% (SD = 0.31). The final categorization accuracy on the test set was 69.83% (see Table 1). As it is reasonable to assume that there may be several appropriate labels for many sentences because articles from different categories can contain similar sentences (e.g., sentences from *Cycling Competition* and *Cyclist*), the top-2 to top-5 accuracies may be more valid estimates of performance.

#### 2.1.2. Word embeddings

Word-level representations were modelled with GloVe vectors (Pennington et al., 2014) trained on Italian words using the code available on https://nlp.stanford.edu/projects/glove/. Vectors of size 300 were trained for a vocabulary of 634,072 words comprising the 596,365 most common words in the Italian Wikipedia (words occurring at least 5 times), and all the words from our training and testing set (so as to have a word vector representation for each word for the models to learn from). All corpus data was pre-processed in the same way as for CNN training before vocabulary extraction. Training was performed on the Italian Wikipedia, extracted from a Wikipedia dump (http://download.wikimedia.org/) using WikipediaExtractor (http://medialab.di.unipi.it/wiki/Wikipedia_Extractor). The total number of tokens in the corpus was 421,369,469. Co-occurrences were counted with a symmetric window of size 15. For GloVe training, alpha was set to 0.75 and x_max to 100 (Pennington et al., 2014). Training was stopped at 25 iterations, yielding a final cost of 0.0060. To test the validity of our embeddings, two tests were performed: (1) we compared embeddings semantic relationships between words with human judgments for 160 basic objects from Giari et al. (2020) and found a strong similarity [*r* = 0.39]; (2) we performed a standard analogy test (Berardi et al., 2015; http://hlt.isti.cnr.it/wordembeddings/) and found good performance: hit rate of 0.41, compared to 0.26 reported by those authors. Word-level sentence meaning was then calculated as the average of the embeddings of the sentence’s constituent words, including all stop words (pronouns, prepositions, determiners, etc.).

### 2.2. Comparing semantic models’ level of abstraction

The purpose of this study is to contrast two levels of sentences’ semantic content: one that has incorporated the combination of words into successively more integrated semantic units to form a semantic ‘gestalt’ or topic (CNN Final Layer) and one that considers the non-integrated average of sentences’ constituent words (average embeddings). One of the advantages of word embeddings is the fact that one can perform mathematical operations on them such as the ones carried out in the analogy test (see Section 2.1.2), but also averaging. Averaging embeddings has often been used in the past to model sentence meaning for a variety of applications such as automatic answer selection (Yu et al., 2014), semantic similarity (Banea et al., 2014) and knowledge completion (Socher et al., 2013). However, while powerful, the averaging operation only treats the sentence as a bag of words: it overlooks word order as well as the differential importance of words within the sentence. It is merely the sum of the parts that constitute the overall meaning of the sentence. In addition, word embeddings are learned through unsupervised learning using the surrounding words and ignores the wider context or general topic of the text. In the current section, we describe the evaluations intended to test whether the CNN model (Final Layer representation) indeed reflected a more relevant topic-level integrated meaning greater than that present in the average embeddings.

We performed two complementary tests to compare the CNN Final Layer to the average embeddings, whose representational dissimilarity matrices (RDMs) correlation yielded *r* = 0.226: (1) we tested whether the CNN model contained more topic-level information (i.e., sentence-level meaning) than the average embeddings using simple nearest centroid classifiers; (2) we qualitatively analysed the structure of semantic distances between sentences using hierarchical clustering, with the hypothesis that distances between categories in the CNN model will reflect more intuitive semantic relationships than the embedding model.

#### 2.2.1. The CNN final layer contains more topic-level semantic information

Two nearest centroid classifiers were constructed, respectively using the average embedding vectors and the CNN Final Layer vectors. Centroids were computed by averaging the vectors across all sentences of each category of the training set (resulting in 64 centroids for each classifier). Each testing set sentence vector was then correlated with each centroid. Classification was done by taking the index of the centroid with the highest correlation Table 1. lists the results of the classifiers.

We observe that the performance using Final Layer vectors is consistently superior to the average embeddings vectors. The Final Layer performance is also close to the actual performance of the model (Table 1).

This result indicates that combinatorial representations in the Final Layer effectively contains more topic-level information than the average embedding model.

#### 2.2.2. Analyzing abstraction with hierarchical clustering across sentence representations

To evaluate whether the CNN model captures the human-intuitive semantic relationships between categories in a better manner than the average embeddings model, we applied Ward hierarchical clustering on the Final Layer and averaged embeddings representations of the sentence stimuli subsequently used in the experiment (see Section 2.2).

**Fig. 2.**
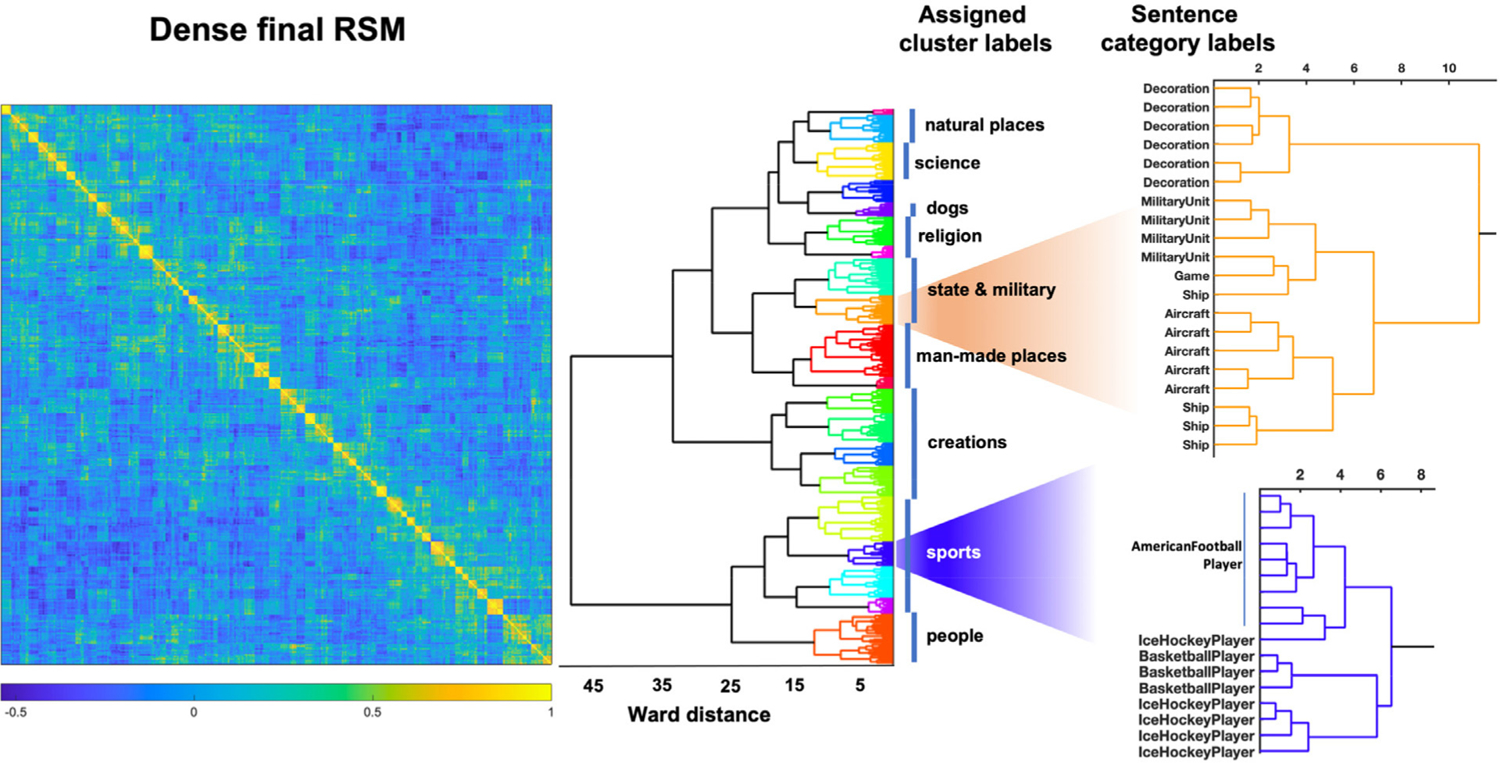
Ward clustering of the sentence stimulus representational similarity matrix (RSM) from CNN Final Layer features. **Left:** RSM of the 384 stimulus sentences using Pearson’s correlation. Each row and column correspond to a sentence; **Centre:** Corresponding dendrogram using Ward hierarchical clustering. The clustering reveals intuitive semantic relationships, in such a way that we were able to assign a representative supra-category label to clusters (*natural places, science*, etc.) which the CNN learned with only the sentence category labels. This was not possible using the average embedding features. **Right:** Details of the full dendrogram from clusters containing sentences related to *Sports* and *State & Military* themes. Sentence category is indicated on the vertical axis. The full tree with labels is available on the Open Science Framework repository (see Section 2.5).

We used hierarchical clustering to see if the similarity between sentences of different categories would capture our intuitions. We found that clustering applied to the CNN model captured human clustering intuitions, with the emergence of natural relationships between categories. Indeed, sentences belonging to related categories tended to cluster together, in a way that allows the assignment of supra-category labels. For instance, sentences belonging to the categories *Basketball Player, American Football Player, Ice Hockey Player* clustered together (see Fig. 2), themselves clustering close to sentences belonging to e.g. the *Cycling Competition* or *Sports Team* categories, effectively forming a sports-themed supra-category. Similarly, sentences associated with vehicles such as *Aircraft* and *Ship* clustered close to *Military Unit* and *Decoration*, forming a military-related supra-category, and so on. The model has therefore learnt between-category semantic structures without this information being fed to it explicitly. By contrast, the clustering results of the average embeddings model showed so little intuitive structure that no supra-category labels could be assigned to the clusters. The full trees with sentence category labels are available for download on the Open Science Framework repository; see Section 2.5.

The cumulative results of the nearest centroid classifier and the hierarchical clustering thus confirm that the CNN model contains more information about sentence category, than the averaged word embeddings, and that this information captures not only topic-level distinctions but also the relationship between them. As an additional control, we tested that the CNN representations outperformed the word-level representation for a benchmark task requiring sentence-level meaning. Because we did not find benchmarks in the Italian language, we used an English version of our model that was trained on 144 Wikipedia categories. We used the semantic textual similarity benchmark (http://ixa2.si.ehu.es/stswiki/index.php/STSbenchmarkCeretl.,2017;), where pairs of sentences were rated according to the similarity of their meaning by humans. Sentences came from news outlets, image captions and online forums, and similarity scores went from 0 (‘The two sentences are completely dissimilar’) to 5 (‘The two sentences are completely equivalent, as they mean the same thing’). The testing dataset that we used comprises 1379 English-language sentence pairs. We found that the CNN representations outperformed the GloVe representations (*r* = 0.54 vs *r* = 0.33) suggesting that the former reflect the global meaning of the sentence better than the word-level representations.

### 2.3. Experiment

#### 2.3.1. Participants

Twenty-five native Italian speakers (female = 11; age range = 20 to 32; mean age = 25.2) took part in the experiment. One participant was excluded due to excessive head movement. Participants gave their informed consent and were compensated for their participation. All procedures were approved by the ethical committee of the University of Trento.

#### 2.3.2. Stimuli preprocessing and selection

Stimuli were algorithmically selected from the Wikipedia corpus to be representative of the model. Sentences with psycholinguistic confounds, such as mean lexical frequency outside the 5 and 95 percentile-range, were excluded using the frequency dictionary from Crepaldi and colleagues (Crepaldi et al., 2015; http://crr.ugent.be/subtlex-it/). Only sentences with length 14 and 15 words were kept. A CNN architecture was partially chosen because it allows quantification of representations at different hierarchical layers and our initial goal was to distinguish brain patterns resembling the Conv1 and Dense2 (Final Layer) layers (see Fig. 1). We performed hierarchical agglomerative clustering with a Ward criterion to form 64 clusters using the vectors at these two hierarchical levels. The top 6 sentences from each cluster (measured by silhouette score) were chosen (i.e., the most representative sentences of each category). Half the sentences were selected for optimal clustering on the Conv1 layer of the model and half on the Final Layer. As a final control, we ran a survey with 4 Native Italian speakers that rated the comprehensibility of the sentences. The 50% more highly rated sentences were selected as stimuli, resulting in 192 sentences representative of each of the two layers (384 in total). The next 24 easiest sentences were selected as distractor sentences for the recognition task (see next section). Despite the strategy to select sentences that were representative of each later, interlayer RDM correlation was very high (*r* = 0.79) and analysis of the differential contribution of Conv1- and Dense2-level representations to observed cortical pattens was deemed unviable.

Examples of sentence stimuli (and their category) translated from Italian are: ‘*The same year, they performed as a pop-rock band in Casa Verdi in Milano*.’ [*Musical Artist*]; ‘*The number of its speakers varies considerably depending on the methodology used to count them*.’ [*University*]; ‘*After the years of conflict, the casino reopened officially on the evening of the 31st of December 1945*.’ [*Building*].

#### 2.3.3. Experimental design and task

The participants’ task was to carefully read the presented sentences while being informed that there would be a subsequent recognition test after each of the 6 runs. In each recognition test, participants indicated whether or not they had seen 8 sentences (48 in total per participant), half of which were distractors. After a practice run with 12 stimuli and a full recognition task performed outside the scanner, participants completed 6 sessions with 64 sentences each. Each sentence was presented word by word for 150 ms per word. Words were presented such that a third of their mass was to the left of fixation and two thirds to the right of fixation (indicated by a red letter). Each sentence lasted on average 2.1 s (max. length = 2.25) and the inter-trial interval was 6 s. Due to equipment failure, responses were not available for 23.6% of trials on average. Average accuracy for recorded responses was 76.8%, well above the 50% chance level, indicating good task-compliance.

#### 2.3.4. fMRI acquisition

We conducted Magnetic resonance imaging (MRI) with a Siemens Prisma 3T scanner (Siemens, Erlangen, Germany) using a 32-channel head coil. 239 vol composed of 68 Anterior Commissure–Posterior Commissure aligned slices were acquired over six runs for each participant. Gradient-echo planar imaging (EPI) sequence parameters were: repetition time TR = 2 s, echo time TE = 28 ms, flip angle = 75°, field of vision (FOV) read = 200 mm, gap size = 0 mm, voxel size = 2 × 2 × 2 mm 3, slices = 68, slice thickness = 2 mm, multi-band acceleration factor = 4. Structural images we acquired using a standard T1-weighted sequence: 192 sagittal slices, FOV = 192 mm, TR = 2.3 s, TE = 2.26 s, flip angle = 9°

#### 2.3.5. fMRI preprocessing

The preprocessing steps were performed using the MATLAB (The Mathworks, Natick, USA) toolbox SPM12 (www.fil.ion.ucl.ac.uk/spm/). Images were realigned for head movement. Slice-acquisition delays were corrected using the middle slice as reference. All images were normalized to the standard SPM12 EPI template (Montreal Neurological Institute MNI stereotactic space), retaining the 2-mm isotropic voxel size, and spatially smoothed using an isotropic Gaussian kernel of 5-mm full-width half-maximum (FWHM). The time series at each voxel for each participant were high pass filtered using a FIR filter of order 80, and cut-off = 0.0156 Hz (64 s).

### 2.4. Data analysis

#### 2.4.1. fMRI searchlight representational similarity analysis

**Fig. 3.**
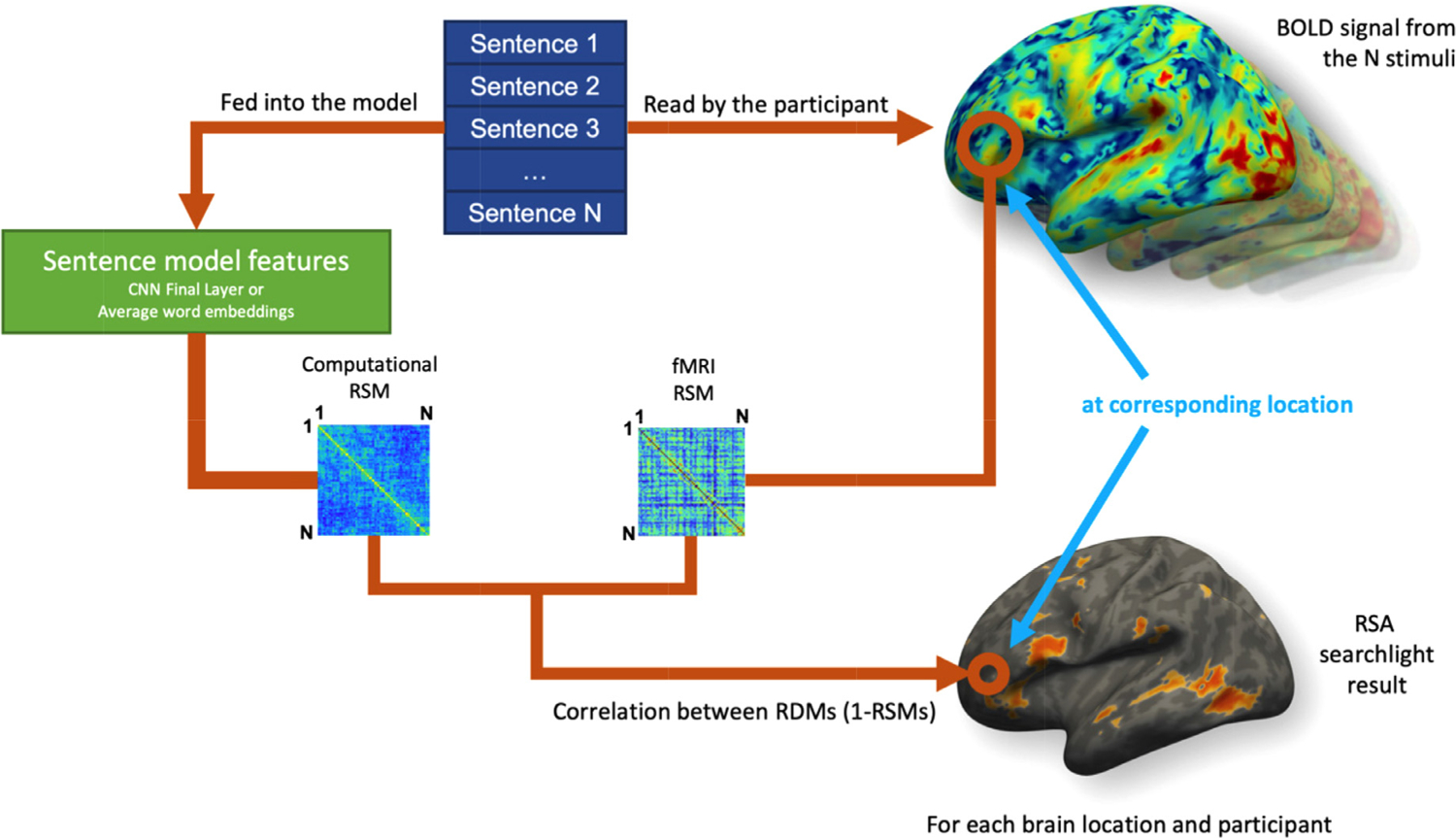
Searchlight representational similarity analysis (RSA) general method. Sentences are modelled using the CNN Final Layer which incorporates semantics produced through the combination of words) and the average word embeddings (which considers the contribution of words individually), and read by humans undergoing fMRI. Representational Similarity matrices (RSMs) are built by computing the pairwise Pearson’s correlation between the feature vectors from the models (averaged word embeddings or the CNN Final Layer), and vectors corresponding to the fMRI voxel values at the searchlight location. RDMs (Representational Dissimilarity Matrices) are then computed from the RSMs and correlated to one another after vectorisation. This process is applied for each searchlight location, model, and participant. Consistent correlations across participants uncover brain regions where neural patterns contain representations of the tested model.

The comparison between fMRI activations and language-derived models was performed using representational similarity analysis (RSA; for a similar approach with images, see Cichy et al., 2016). Searchlight RSA was implemented using the CoSMoMVPA MATLAB toolbox (Oosterhof et al., 2016). RSA was performed twice on the whole brain using either representations of the sentences from average word embeddings or the Final Layer of the CNN. The two representational similarity matrices (RSM) were built by calculating the similarity between each pair of sentences using the average word embeddings or the Final Layer of the CNN (Pearson’s correlations). This resulted in two 384 × 384 RSMs reflecting the similarity of each sentence pair for embedding and CNN models, from which two RDMs (1-RSMs) were calculated. These RDMs were then used in subsequent brain-model RSA (see Fig. 3). The searchlight RSAs (radius 8 mm) were performed across the whole brain of each participant. At each searchlight location, an fMRI RDM was built using Pearson’s correlations after demeaning the data. The correlation between the vectorised sentence RDMs (derived from the embeddings or the CNN Final layer) and the vectorised fMRI RDMs was then calculated using Pearson’s correlation. The correlation value was reported at the centre voxel of the sphere. Finally, the correlation maps were Fischer-transformed and smoothed using a kernel of 12-mm FWHM.

#### 2.4.2. ROI-based RSA

Eight cortical regions of interest (ROIs) known to be involved in semantic processing (Binder et al., 2009) were selected on both hemispheres: the angular gyrus (AG), the precuneus, the posterior middle temporal gyrus (pMTG), the lateral anterior temporal lobe (latATL), the ventral temporal cortex (VTC), the inferior frontal gyrus (IFG), the dorsomedial prefrontal cortex (dmPFC) and the ventromedial prefrontal cortex (vmPFC). Regions were selected using the Brainnetome Atlas (Fan et al., 2016) available at https://atlas.brainnetome.org. Additional MNI coordinate constraints were defined for the VTC and the latATL. The latATL ROI excludes all Brainnetome subregions of the superior anterior gyrus (with the exception of subrostral area 22 covering the anterior superior temporal sulcus) and covers BA21. ROI shapes were smoothed by applying a dilation, followed by an erosion using a spherical kernel of size 5 Table 2. indicates the subregions and MNI coordinate constrains defining each ROI, its respective size and centre of mass location, while Fig. 5 A schematizes their locations. RSAs were performed using all the voxels of each ROI.

#### 2.4.3. Statistical analysis

**Fig. 4.**
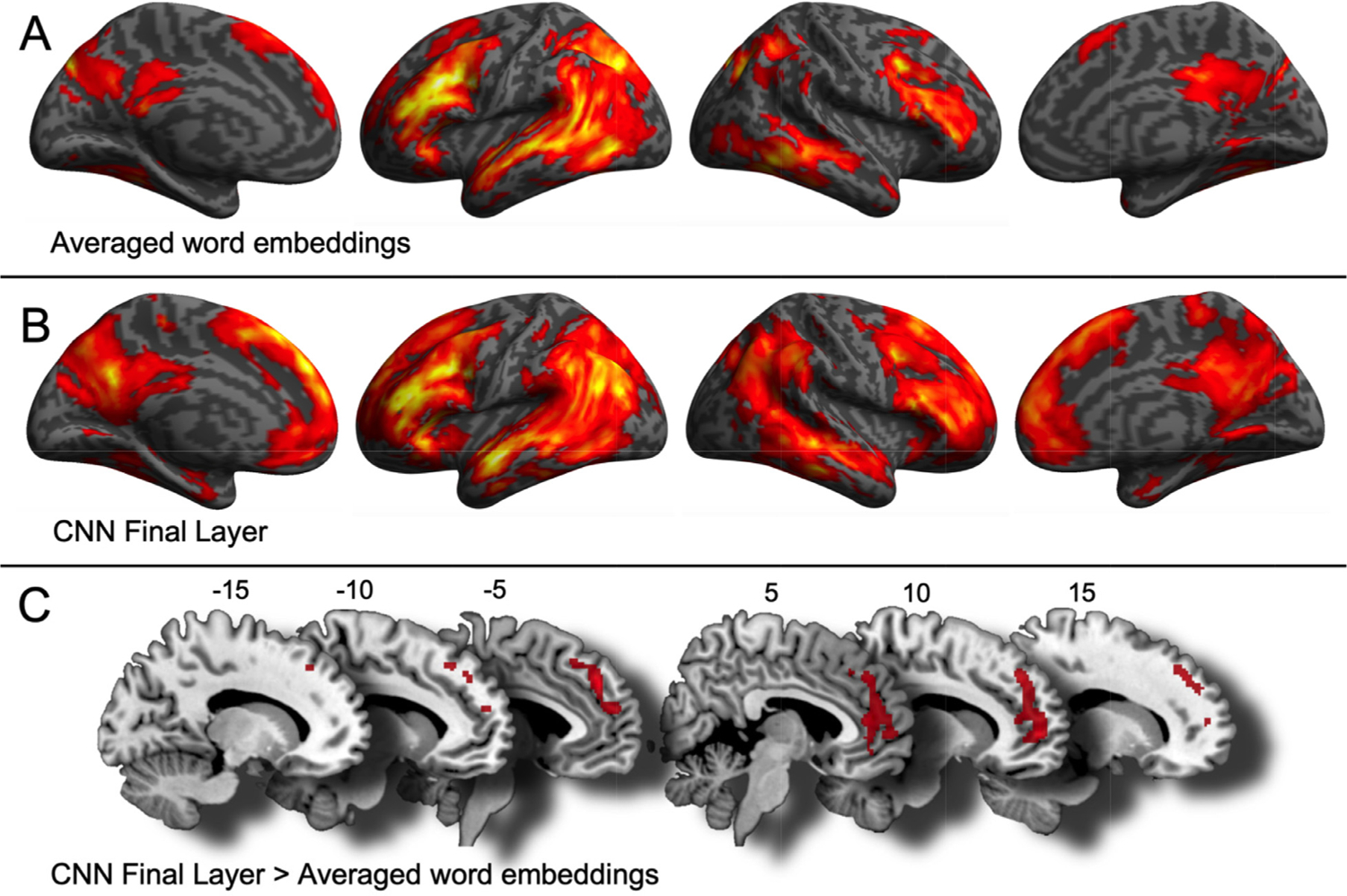
Representational similarity analysis searchlight (RSA) results. Areas explained by **(A)** Averaged word embeddings (word-level semantic meaning), and **(B)** CNN Final Layer (incorporating combinatorial semantic meaning). Surface projection of the T maps from the general linear model. **(C)** Areas better explained by the CNN Final Layer than the averaged word embeddings. Sagittal slices at the indicated X-axis MNI coordinates. All locations shown are significant at *p* < 0.001, cluster-corrected.

Statistical analyses on searchlight RSA output were performed using SPM12. To find regions that were significantly explained by the averaged embeddings and the CNN Final Layer, the correlation maps from the 24 participants were fed into a one-factor two-level general linear model with subject-specific constants. The contrast between Final Layer and embeddings was evaluated using one-tailed paired-sample t-tests. Reported results present locations where *p* < 0.001, cluster-corrected at *p* < 0.05 using family-wise error (FWE). Visualizations were performed using SPM12 rendering (Fig. 4 A and 4 B) and MRIcron (Fig. 4 C). For ROI analysis, one-tailed paired-sample t-tests with Bonferroni multiple-comparisons correction were used to compare RSA outputs between the CNN Final Layer and the average embeddings models.

### 2.5. Data and code availability

**Fig. 5.**
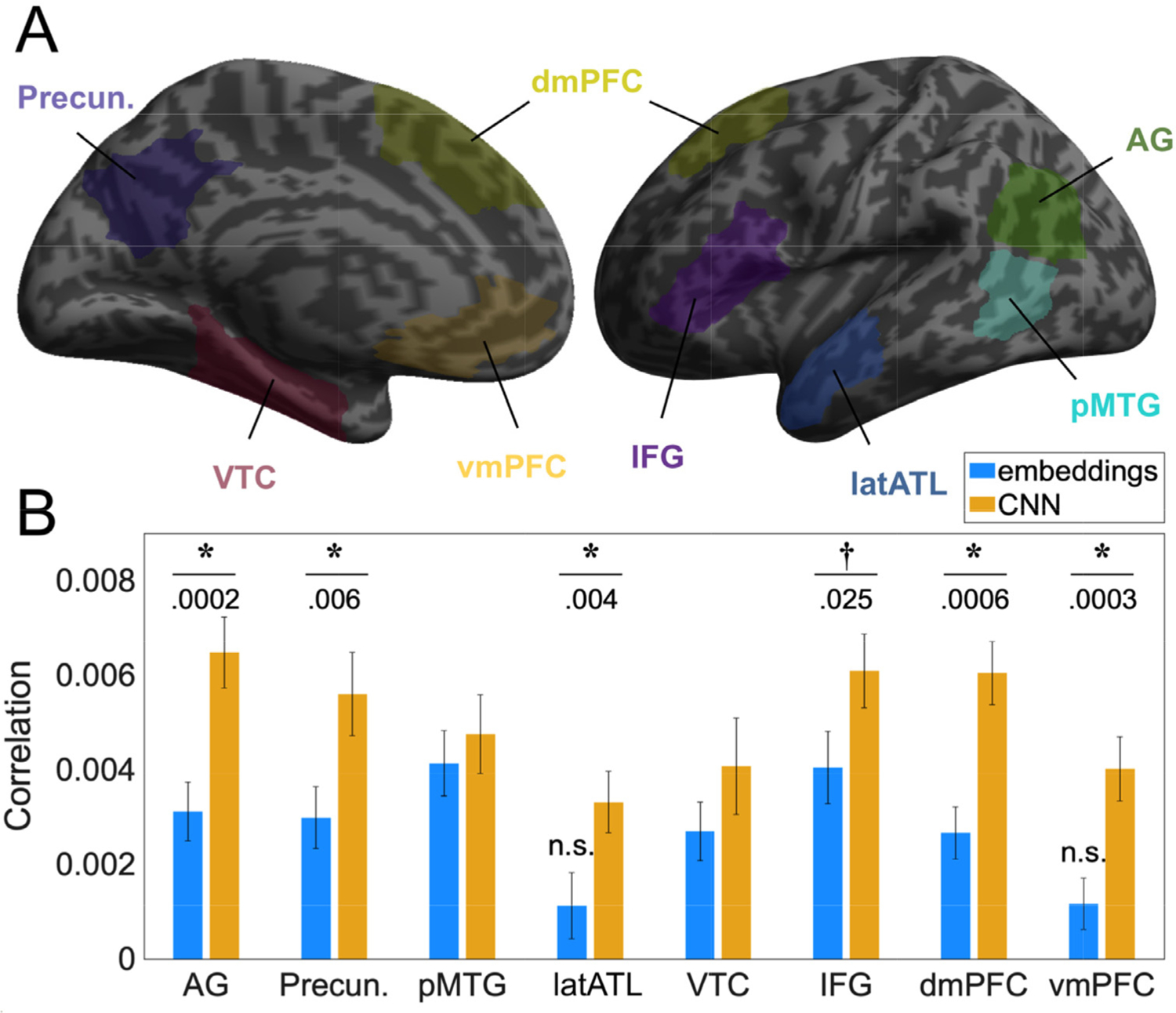
Region of interest (ROI) analysis**. (A)** Schematic localisation of the cortical ROIs covering the semantic network. All ROIs are bilateral Table 2. provides details on the location, size and regions of each ROI. **(B)** Mean RSA results for each ROI and model (CNN Final Layer and average embeddings). Mean correlation across participants +/− standard errors (as described in (Cousineau, 2005). Differences between models (paired t-tests): [*] Significant with Bonferroni correction; [†] Significant without correction only. Departure from zero (one-sample t-tests): [n.s.] non-significant.

Participant-level RSA data for the searchlight (Fig. 4), ROI-based analyses (Fig. 5) and full dendrograms with labels (Fig. 2) are available on the Open Science Framework repository: https://osf.io/95ftn/?view_only=9a1a085583544c3eac44d1c75870599c. Datasets and code will be made available upon request.

## 3. Results

### 3.1. Whole-brain searchlight analyses

Participants’ brain activity was recorded while reading sentences. A whole-brain searchlight RSA strategy was applied to identify regions whose patterns were best captured by the CNN-based and word-level models (see Fig. 3 for a summary). RSA revealed that word-level representations were widespread across the cortical surface (Fig. 4 A). They were more left-lateralised and regions included left precentral gyrus, bilateral IFG, bilateral occipito-temporal regions, bilateral precuneus and left SMA and dmPFC. Sentence topic-level representations (Fig. 4 B) converged on word-level representation but encompassed greater sections of the precuneus, and additionally included the dmPFC, vmPFC and the right AG. To formally test which brain areas contained information more consistent with combinatorial semantic representations rather than word-level sentence representations, we performed a paired *t*-test, which revealed significantly greater information capture by the sentence-level model (CNN Final Layer representations) in the vm/dmPFC (see Fig. 4 C, Table 3).

We did not find any region significantly better explained by the embedding than CNN model.

### 3.2. ROI-based RSAs

In addition to the whole-brain analyses, we sought to investigate differences between CNN and word-level meaning across the main regions of the semantic network. For this purpose, we performed RSAs in 8 ROIs in each hemisphere defined from (Binder et al., 2009) (see Fig 5 A and methods Section 2.4.2.).

As a control, we tested whether we could observe a significant RSA result using the word embeddings in the left ventral anterior temporal lobe. We defined an additional ROI from Jackson et al. (2016) using the mSTG, rostral parahippocampal gyrus and area TI Brainnetome subregions (with respective Brainnetome indices 69, 109 and 117). We found a significant positive correlation with the embedding model: t(23) = 2.89, *p* = 0.008 [*m* = 0.0026; std = 0.0044]. This replicates previous reports (Bruffaerts et al., 2013) and provides confidence in the validity of the experimental paradigm and the word-level model.

As a preliminary analysis, we tested the lateralization of the embeddings and CNN Final Layer representations in the semantic network, as well as differences in lateralization between the two. To this end, we entered RSA results for the ROIs situated laterally (AG, pMTG, latATL and IFG) into a Hemisphere (Left, Right) × ROI (AG, pMTG, latATL, IFG) × Model (embeddings, Final Layer) repeated measures ANOVA. We found that while both models explained brain patterns better on the left hemisphere than on the right hemisphere [F(1,23) = 9.589, *p* = 0.005], there was no interaction of Hemisphere with Model, ROI and Model × ROI (all Fs < 1). This was confirmed by 4 ROI-specific Hemisphere × Model (2 × 2) ANOVAs, all yielding non-significant interactions (Fs < 1). As a consequence, we performed RSAs on the ROIs collapsed across hemispheres.

The CNN model predicted neural patterns in all ROIs (one-tailed t-tests on correlation values: all ts > 2.8 and all p_uncorr_ < 0.004, surviving Bonferroni correction across ROIs with adjusted alpha = 0.0063), except for the latATL and the vmPFC whose correlations with the average embeddings model did not significantly depart from zero even at uncorrected level: respectively t(23) = 1.05, p_uncorr_ = 0.152 and t(23) = 1.64, p_uncorr_ = 0.056 (indicated as *n.s*. in Fig. 5 B).

In the critical analysis, we compared the information present in the CNN and embeddings derived representational spaces. Specifically, one-tailed paired t-tests were performed on each ROI, testing in which regions the CNN model better explained brain patterns than the embeddings model (see Fig. 5 B). CNN-derived representational spaces better explained brain data in the AG [t(23) = 4.20, p_uncorr_ = 0.0002, *d* = 0.86], precuneus [t(23) = 2.73, p_uncorr_ = 0.006, *d* = 0.56], latATL [t(23) = 2.92, p_uncorr_ = 0.004, *d* = 0.60], dmPFC [t(23) = 3.67, p_uncorr_ = 0.0006, *d* = 0.75] and vmPFC [t(23) = 3.95, p_uncorr_ = 0.0003, *d* = 0.81], surviving Bonferroni correction. Uncorrected differences were also found in the IFG [t(23) = 2.07, p_uncorr_ = 0.025, *d* = 0.42]. No significant differences were found for the pMTG [t(23) = 0.55, p_uncorr_ = 0.29, *d* = 0.11] and VTC [t(23) = 1.22, p_uncorr_ = 0.12, *d* = 0.25]. These results are consistent with medium effect sizes in latATL and the precuneus and large effect sizes in AG, vmPFC and dmPFC.

To assess whether inclusion of bilateral regions in the ROI analysis diluted differences between the two models, the RSA was also performed on the left hemisphere for lateral ROIs (AG, pMTG, latATL and IFG). We observed a drop in statistical power but found similar results. Significant differences were present in the AG [t(23) = 3.12, p_uncorr_ = 0.002] with Bonferroni-correct alpha = 0.0125, and at uncorrected levels in the latATL [t(23) = 2.18, p_uncorr_ = 0.020] and below the 0.05 threshold in the IFG [t(23) = 1.65, p_uncorr_ = 0.057]. As in the bilateral-ROI analysis, no significant differences were evident in the pMTG [t(23) = 0.42, p_uncorr_ = 0.34].

### 3.3. Comparison with other models

The CNN was trained specifically to categorize sentences into Wikipedia categories in a supervised manner, while the embeddings were trained to form a general model of word meaning in an unsupervised manner. To ensure that observed differences were not attributable to the fact that the word-level embeddings were uninformed about Wikipedia category while the sentence topic-level CNN was informed, we performed a supplementary analysis. We built an category-informed word-level model using the tf–idf (term frequency, inverse document frequency Salton and Buckley, 1988;) features of the words. This model determines the importance of each word in distinguishing the 64 categories and these were averaged into a vector for each sentence. Each word was represented by a tf–idf vector of size 64. Sentences were represented by the average tf–idf across all of its constituent words. An RDM was built from these tf–idf sentence representations, and then correlated to the fMRI-RDM of each ROI. Performing the test on the 6 significant ROIs using the embeddings (adjusted alpha = 0.0083), we found that the CNN model continued to better explain neural patterns in the AG [t(23) = 2.60; p_uncorr_ = 0.0082], IFG [t(23) = 3.61; p_uncorr_ = 0.0007], dmPFC [t(23) = 2.95; p_uncorr_ = 0.0036] and vmPFC [t(23) = 3.31; p_uncorr_ = 0.0015], but not in the latATL [t(23) = 2.48; p_uncorr_ = 0.01] and the precuneus [t(23) = 1.62; p_uncorr_ = 0.059].

To assess how our model derived from a CNN designed to capture specific semantic categories compared with pre-trained state-of-the-art sentence encoders, we compared results using the Multilingual Universal Sentence Encoder Large V3 (MUSEL3; Google LLC, CA, USA Chidambaram et al., 2019;; https://tfhub.dev/google/universal-sentence-encoder-multilingual-large/3). No significant difference was observed between CNN and MUSEL3 models, with the CNN model showing only a trend towards better explaining neural representations. This tendency was also evident when contrasting MUSEL3 with the embedding model where there was no evidence that MUSEL3 better explained neural representational spaces better than the averaged embeddings after correction for multiple comparisons [AG: t(23) = 2.22, p_uncorr_ = 0.018; precuneus: t(23) = 2.68, p_uncorr_ = 0.007; pMTG: t(23) = 0.62, p_uncorr_ = 0.27; latATL: t(23) = 2.4, p_uncorr_ = 0.012; VTC: t(23) = 0.81, p_uncorr_ = 0.21; IFG: t(23) = 1.98, p_uncorr_ = 0.030; dmPFC: t(23) = 1.96, p_uncorr_ = 0.031; vmPFC: t(23) = 1.63, p_uncorr_ = 0.059].

CNNs create representations of sentences through the hierarchical combination of tokens into successively larger chunks and units of semantic meaning. In a post hoc control analysis to assess the importance of this localised context, we constructed a fully connected multilayer perceptron (MLP). Using the same Wikipedia training data, we set the inputs to the averaged sentence embeddings and used the scikit-learn library (parameters following gridsearch: 3 layers with 300, 512 and 128 nodes, followed by softmax layer; Alpha: 0.1; Batch size: 256; Learning rate init: 0.001; Activation: tanh; Solver: adam). Averaging word embeddings does not take word order into account but using the MLP allows these representations to be transformed in order to predict a label (the topic). Therefore, due to the inputs and fully connected nature of the MLP, it does not contain hierarchical information about context, but does share the training goal of distinguishing the 64 Wikipedia topics (test-set accuracy 74.72%).

Compared to averaged word embeddings, MLP-derived representational spaces better explained brain data in the AG [t(23) = 2.85, puncorr = 0.005;] and dmPFC [t(23) = 2.94, puncorr = 0.004], surviving Bonferroni correction. Uncorrected differences were found in the precuneus [t(23) = 2.46, puncorr = 0.011],; latATL [t(23) = 1.99, puncorr = 0.029], IFG [t(23) = 2.09, puncorr = 0.024] and vmPFC [t(23) = 2.15, puncorr = 0.021]. No significant differences were found in pMTG [t(23) = 1.19, puncorr = 0.123] or VTC [t(23) = 1.28, puncorr = 0.106]. This pattern of results is consistent with those observed when comparing averaged embedding to the CNN and no significant differences were present between the amount of neural variance accounted for by CNN and MLP models. Overall, the results provide no evidence that localised context present in CNNs aids in the modelling of neural representational spaces and suggests that topic-level training objectives best accounts for the convergence between computational and neural models of sentence meaning.

## 4. Discussion

The goal of this study was to identify brain regions that represent the topic-level meaning of sentences. To this end, we trained a CNN to distinguish between sentences drawn from 64 broad semantic categories present in Wikipedia. The model learned not only to effectively identify these topic categories but also the natural relationship between them. These overarching similarity relations emerged even though the model did not know the relatedness between categories *a priori*. We then compared representational similarity spaces of 384 sentences using the Final Layer of the CNN (sentence-level meaning) and average word embeddings (word-level meaning) to the fMRI activity from reading the same sentences.

We found that neural patterns significantly explained by both sentence topic- and word-level models were broadly distributed, with a high degree of overlap and stronger representations in the left hemisphere. The overlapping distribution of these effects indicates that the cortical regions contributing to sentence topic and word-level meaning are not fully discrete and share common cortical substrates. The distributions of both sentence topic- and word-level brain maps is highly consistent with the anatomical location of the semantic system (Binder et al., 2009), as well as those regions showing sensitivity to single object-concepts in the multivariate pattern of the response (Fairhall and Caramazza, 2013; see also Pereira et al., 2018). In a control analysis we found that such word-level concept representation extended to the left medial temporal lobe (Bruffaerts et al., 2013). The fact that both word-level and sentence topic-level representations of sentences explained activation patterns in these regions underscores the importance of individual tokens (words) in the way in which semantic knowledge is represented in the brain.

The critical contrast between these two models revealed stronger topic-level than word-level representations in ventral and dorsal components of the medial PFC. This whole-brain contrast was supported by an *a priori* anatomical ROI analysis encompassing the key nodes of the semantic system (Binder et al., 2009). This revealed stronger sentence topic-level (vs. word-level) stimuli representations not only in the vmPFC and dmPFC but additionally in the AG, latATL and precuneus. These differences cannot simply be attributed to the specialized topic-related information that is not present at the individual word level. The contrast of our CNN sentence-topic model with the tf–idf word-level model, designed to determine the importance of each word into distinguishing the model categories, yielded comparable results, except for the latATL and precuneus which did not survive the multiple comparison correction. Although mechanisms or transformations differ between the brain’s semantic system and the CNN, this collectively provides powerful external validation for the enhanced capacity of deep neural networks to capture human-like representational spaces (see also Jain and Huth, 2018). Importantly, our results also reveal how information is represented across the semantic system, emphasising the role of the medial PFC, AG, latATL and precuneus in the representation of sentence meaning at the topic level.

A multilayer perceptron was used to model topics with an architectural complexity closer to that of the CNN. Here, we observed similar performance between the MLP and the CNN. Unlike the tf-idf model, which focusses on individual words within the topic training set and is uninformed about sentence membership, the input into the MLP was the averaged word embeddings for each sentence. It is possible that the architecture of the MLP, coupled with the algebraic properties of word embeddings discussed in Section 2.1.2., may render this model sensitive to patterns in the occurrence of words, or semantic themes, within sentences. For this reason, it is uncertain if context at the level of word occurrence within the same sentence may have contributed to the ability of this model to explain neural data. On the other hand, these results suggest that the localised context afforded by the hierarchical nature of CNN models and the flexible construction of combinatorial meaning at progressively larger scales, is not critical to the capacity of these models to explain neural representational patterns and we have no evidence that combinatory semantic representations contribute to the observed cortical representation.

In the following paragraphs, we discuss our ROI-based results by referring to relevant functions that each region has been associated with. Informal reverse-inference type reasoning can be problematic, in particular when the inferences are stated as facts rather than conjectures, but it can also provide useful insight about the role of the regions and the function investigated (Poldrack, 2011 Young and Saxe, 2009;).

The most reliable difference in sentence topic-level versus word-level meaning was found in the medial PFC. The dmPFC has been proposed to support self-guided, goal-directed retrieval of conceptual knowledge (Binder et al., 2009) and the vmPFC linked to the affective significance and reward value of concepts (Binder et al., 2009 Ferstl et al., 2005; Jackson et al., 2019;). At the same time, these medial PFC regions have also been implicated in a more general role in the combination of concepts, ranging from the combination of noun-noun compound words (Forgács et al., 2012 Graves et al., 2010;) to the construction of meaning over long narrative timescales (paragraphs or longer) (Lerner et al., 2011 Yarkoni et al., 2008;). Our results provide further support for the involvement of medial PFC in the representation of broad sentence meaning at the topic level.

Topic-level meaning was also better represented than context-free word-level meaning in the AG, the latATL and the precuneus. The AG has typically been associated with a range of semantic operations (Binder et al., 2009 Price et al., 2015; A.R. 2016;), including general ‘taxonomic’ associations between concepts that are formed through confluence of shared features (e.g., *apple* and *pear*) but particularly in thematic associations between pairs of concepts (e.g., *mouse* and *cheese* Boylan et al., 2015; Kalénine et al., 2009; Schwartz et al., 2011;). These thematic associations, that reflect the more flexible combination of unlike concepts, may rely on shared neural substrates or computations necessary for topic representation. Indeed, the AG has been recently identified as a key structure for the representation of meaning at the level of paragraphs (Branzi et al., 2021). The precuneus is one of the most consistently reported brain regions in studies of semantic processing (Binder et al., 2009). Its recruitment in semantic tasks has previously been attributed to the incidental retrieval of episodic memories (Binder et al., 2009 Gobbini and Haxby, 2007;). However, this region is also activated when people access semantic properties about animals (Binder et al., 1999), or the nationality or occupation of famous people (Aglinskas and Fairhall, 2019 Fairhall et al., 2014;) and for sentences containing two object-concepts from different (compared to the same) object domains (Rabini et al., 2021). Moreover, RSA has shown that representational spaces in voxel level patterns in the precuneus conform to semantic representational spaces for single concepts (Fairhall and Caramazza, 2013). Collectively, this suggests a role of the precuneus in semantic representation that extends beyond that of an episodic by- product of semantic access. The present result supports that assertion and extends it to show a role not only in the representation of individual concepts but also in the representation of more general contextual meaning.

The ATL has long been identified as a critical region for semantic processing, largely on the basis of neuropsychological studies of lesions, semantic dementia and herpes encephalitis (Patterson et al., 2007), but also functional neuroimaging data (Visser et al., 2010). Source reconstruction of magnetoencephalography data also indicate a role of this region in sentence composition (Brennan and Pylkkänen, 2012, 2017). Interestingly, there is a lack of evidence for single concept representations in the lateral ATL using whole-brain functional neuroimaging on healthy participants (Bruffaerts et al., 2013 Devereux et al., 2013; Fairhall and Caramazza, 2013;). A similar pattern is reflected here, where the word-level models of sentence representations (averaged embeddings) failed to explain representational spaces within this region. On the other hand, ATL has been previously associated with the formation of composite concepts important for sentence comprehension (Frankland and Greene, 2020 Friederici, 2011; Pallier et al., 2011;; see also Chadwick et al., 2016). Our pattern of results suggests the lateral ATL may have a particular role in the representation of the ensemble, topic-level, semantic content of sentences.

It is notable that the four identified regions (lateral ATL, medial PFC, AG and precuneus) belong to the default mode network (DMN; c.f Buckner et al., 2008.). The functions of this network relate not only to eponymous ‘default-mode’, task-deactivated states (Raichle et al., 2001) but include a broad range of internalised integrative cognitive processes: context integration, episodic memory and mental time travel (Keidel et al., 2018 Schacter and Addis, 2007; Viard et al., 2011;), social cognition (Greene et al., 2001 Van Overwalle, 2009;, 2011), as well as general semantic knowledge (Binder et al., 2009 Fairhall and Caramazza, 2013;; see Spreng et al., 2009 for a review). Unlike the processing of a single word that may be directly available as stored memory, the comprehension of a sentence most frequently requires the construction of new meaning and the extraction of a coarse context meaning or topic, which shares many of the computational demands common with these high-level internally-driven and integrative processes. The overlapping involvement of the DMN across these varied forms of cognition may reflect an intersecting role in the flexible integration of information. In this way, it may be that, rather than a single region sitting atop the conceptual processing hierarchy, this operation is supported in a distributed fashion by multiple nodes of the default mode system. A similar argument was recently put forward (Frankland and Greene, 2020) with the authors proposing that the conceptual combinations that underlie the representation of the meaning of sentences also underlie the human capacity to internally generate complex thought through the Language of Thought model (Fodor, 1975). Under such a postulate, the integrated representations of sentence topic-level meaning reported in this study may additionally form a putative basis for higher-level generative thought. While these DMN regions may play a central role in the representation of integrated topic-level meaning, this does not exclude the possibility that it may accomplish this via coordination with brain areas specialised for access to specific kinds of object or in the flexible access to different kinds of content (e.g. geographic knowledge about food Fairhall, 2020;).

Some regions frequently implicated in sentence processing (Fedorenko et al., 2010) were not reported as representing topic-level sentence meaning with our RSA approach. It is through the syntactic-semantic principles of language that single words and individual concepts are combined into more complex meaning. Language regions, defined as those that result from the contrast of sentences (which contain semantics, morphology, and syntax) with sequences of non-words (which do not), have shown to be stable within and across individuals, visual and auditory modalities, and languages (Fedorenko et al., 2010; for a discussion, see Fedorenko, 2014). The present result may highlight different forms of cortical activity uncovered using univariate approaches (classically used to identify language regions) and multivariate measures of information representation (i.e., RSA). The univariate increase in blood oxygenation level dependant (BOLD) signal as a function of integrative demand may, in particular, uncover integration *processes* (e.g. syntactic processing, syntactic-semantic integration). For instance, Pallier and colleagues (Pallier et al., 2011) observed a parametric increase in activity with the number of linguistic constituents within a series of words or pseudowords in the left IFG, and extending from the anterior temporal lobe to the angular gyrus. This approach is in contrast to the use of RSA in the present study, which finds activity patterns that conform to semantic informational structures for stimuli balanced on the overall level of integrative demand. Regions like the ATL and angular gyrus that show both a representation of sentence-level topic meaning (present study) and a parametric sensitivity to integrative demand (Pallier et al., 2011) potentially play a role in both the process of sentence integration/composition as well as representing the result of that process – the topic-level meaning of the sentences. Regions like the mPFC and precuneus that represent the topic-level meaning of the sentence but are not sensitive to integrative demand, may play a role predominantly in representing the ensemble sentence meaning rather than in its construction. Finally, regions like the IFG, which show weak or no representation of topic-level meaning but that are modulated by integrative demand (Pallier et al., 2011) may play a role in the integrative process but not in the final representation of that process. This dissociation in role is further supported by the finding that, unlike the ATL or AG, the parametric modulation of response in the IFG is comparable for meaningful and meaningless jabberwocky sentences (Pallier et al., 2011), underscoring the role of this region in the integration process even when meaning is absent. This is consistent with the broader role of the IFG in semantic control (Binder et al., 2009 Lambon Ralph et al., 2017; Martin and Chao, 2001; Thompson-Schill et al., 1997;), syntactic-semantic integration (Friederici, 2011) and the domain-general role of this region in short-term memory, on which sentence comprehension relies (Rogalsky and Hickok, 2011).

A similar distinction may explain in particular why the pMTG did not show reliably stronger sentence topic-level than word-level representations. However, while the pMTG has also been implicated in semantic control (Lambon Ralph et al., 2017 Noonan et al., 2013;), it has additionally been implicated in the semantic representation of single concepts (Devereux et al., 2013 Fairhall and Caramazza, 2013;), suggesting a complex role in both representation and control. In particular, it has been suggested that interactions between the IFG and the pMTG allow sustained representations in short-term memory throughout sentence processing, for integration into the overall context (Lyu et al., 2019 Noonan et al., 2013; Turken and Dronkers, 2011;). The lack of observed difference between models in the pMTG may therefore be explained by its main involvement in semantic control and single concept representation.

For comparison, we compared the Wikipedia trained model to a state-of-the-art sentence level meaning encoder, MUSEL3. The MUSEL3 encoder situates itself between the GloVe and the CNN (without any significant differences with the other two models). The (non-significantly) better correlation of the CNN with brain data may be explained by the fact that the CNN model considers more the overarching meaning of the sentence, while the MUSEL3 is more general, as it was trained on an several tasks (conversational response prediction, quick-thought, natural language inference). It could also be due to the particular set of sentences used in the experiment, which were selected to emphasise broad thematic differences rather than subtle changes in meaning between similar sentences.

Indeed, one limitation of our study is that the stimulus-selection process was designed to maximally differentiate topics and identify brain activity that captured the relationship between these topics. One potential side effect of this approach is that the resulting RDMs may be maximized to detect differences at the sentence/CNN level rather than the word/averaged-embeddings level. Future work employing stimuli that maximize information in word-level RDM models may highlight cortical regions that better represent meaning at the level of individual words. A further potential consideration is that the averaging of word embeddings may result in a loss of information about the specific words composing the sentence. One of the advantages of word embeddings is the fact that one can perform mathematical operations on them. In particular, averaging embeddings has often been used in the past to model sentence meaning for a variety of natural language processing applications (e.g., Banea et al., 2014 Socher et al., 2013; Yu et al., 2014;). Furthermore, it has successfully been used to model brain activity. In particular, Pereira et al. (2018) were able to predict average embeddings from the BOLD signal, which further underlines the relevance of this model. These past works validate the relevance of average word embeddings representational space as a model for brain representations. Our work intends to go further than the averaging model that treats words independently.

In sum, we mapped two types of sentence representations onto the cortex using RSA: word-level representational spaces (derived from averaged word embeddings, modelling individual word-meaning independently of the topic-level meaning of the sentence) and sentence topic-level representational spaces that consider the combination of words into a higher-level semantic unit (from the CNN Final Layer). This higher-level representation can be seen as a topic-level representation, as the CNN is trained on broad categories and thus will not be sensitive to subtle semantic and syntactic changes. While both models explained neural patterns in broad overlapping areas of the cortex, indicating a shared reliance on common cortical regions, the CNN model more fully captured cortical representational spaces of sentences in medial PFC, the precuneus, the AG and the lateral ATL. Analyses exploiting an MLP model using the averaged word embeddings as input supported the role of topic-level meaning - rather than integrated meaning built from fine-grained contextual information within the sentence - in explaining neural representational spaces in the regions. The location of these regions provides insight into how these representations manifest in the brain. Specifically, the location across multiple regions within the DMN is consistent with: a) a broader role of this network, associated with other high-level internally-driven processes, in the representation of broad thematic information; b) the distributed representation of topic-level meaning across this network rather than being confined to a single node or hub; c) a potential distinction between regions primarily involved in the representation of meaning within the DMN and language-related regions outside of this network that are involved in the processes underlying the integration of sentences.

## Figures and Tables

**Table 1:** Nearest centroid classifiers results, compared to chance level and full model output accuracy (%) for CNN and MLP models.

	Top 1	Top 2	Top 3	Top4	Top 5
**Chance level**	1.6	3.1	4.7	6.3	7.8
**Embeddings nearest centroid classifier**	45.4	59.3	66.8	71.7	75.3
**CNN Final Layer nearest centroid classifier**	69.0	80.4	85.5	88.6	90.6
**MLP Final Layer nearest centroid classifier**	68.8	80.9	86.5	89.8	91.9
**CNN output category**	69.8	81.1	86.4	89.4	91.4
**MLP output category**	74.7	86.1	90.8	93.3	94.9

**Table 2:** Details of the region of interests (ROIs) covering the main regions of the semantic system. Each ROI was constructed using subregions of the Brainnetome atlas (Fan et al., 2016). For each ROI, we indicate number of voxels and centre of mass location. (AG = angular gyrus; Precun. = precuneus; pMTG = posterior middle temporal gyrus; latATL = lateral anterior temporal lobe; VTC = ventral temporal cortex; IFG = inferior temporal gyrus; dmPFC = dorsomedial prefrontal cortex; vmPFC = ventromedial prefrontal cortex.).

ROI	Brainnetome atlas indices of included subregions (both hemispheres)	size in 2 × 2 × 2-mm-voxels l and r hemisphere		centre of mass mni coordinates l and r hemisphere	
AG	143, 144	1597	1383	[−47 −65 26]	[53 −54 25]
Precun.	151–154	1794	2147	[−10 −61 29]	[12 −59 29]
pMTG	85, 86, 97, 98	1083	1133	[−57 −59 0]	[58 −55 −1]
latATL	79–84, 87, 88 ∩ y_MNI_ ≥ −23	2211	2427	[−59 −18 −11]	[60 −20 −10]
VTC	103–120 ∩ −47 ≤ y_MNI_ ≤ −7	1849	1928	[−31 −28 −24]	[32 −27 −24]
IFG	29–40	2437	2556	[−47 24 9]	[48 25 10]
dmPFC	1–4, 11, 12	2782	2943	[−10 25 49]	[11 27 47]
vmPFC	41, 42, 49, 50, 187, 188	2190	1963	[−8 33 −11]	[7 34 −9]

**Table 3:** Contrast between sentence-level and word-level representations, from searchlight representational similarity analyses (RSA). Stronger combinatorial than word-level representations constituted one cluster in the medial PFC (see Fig. 4 C). Significance and extent of the cluster, and significance, t-value and location of peaks. (dmPFC = dorsomedial prefrontal cortex; vmPFC = ventromedial prefrontal cortex.).

	Cluster		Peaks				
Locations	p_FWE-corr_	Extent	p_FWE-corr_	t	X	Y	Z
dmPFC	<0.001	1052	0.131	5.71	−6	44	36
vmPFC			0.253	5.33	10	54	2

## Data Availability

Participant-level RSA data for the searchlight (Fig. 4) and ROI-based analyses (Fig. 5) and full dendrograms with labels (Fig. 2) are available on the Open Science Framework repository: https://osf.io/95ftn/?view_only=9a1a085583544c3eac44d1c75870599c. Datasets and code will be made available upon request.
